# Use of health information from social media and health self-efficacy: a cross-sectional study

**DOI:** 10.3389/fpubh.2026.1821668

**Published:** 2026-06-15

**Authors:** Xiaoyu Liu, Vijaya Tamla Rai, Yi Yin

**Affiliations:** 1 College for Public Health and Social Justice, Saint Louis University, Saint Louis, MO, United States; 2 Department of Sociology & Anthropology, Montana State University, Bozeman, MT, United States; 3 Department of Behavioral Science, Utah Valley University, Orem, UT, United States

**Keywords:** digital health literacy, health information seeking, health management, health self-efficacy, HINTS 6, misinformation, social media

## Abstract

**Background:**

Social media has become a common source for health information, yet the relationship between users’ experiences with this form of digital health information and health self-efficacy remains poorly understood. This study investigates how the reported reliance on and perceived evaluative difficulty of social-media-based health information are associated with self-efficacy levels in a misinformation-prone environment.

**Methods:**

This cross-sectional study analyzed data from the 2022 Health Information National Trends Survey (HINTS 6), a nationally representative sample of U. S. adults (*N* = 4,352). Binomial logistic regression was used to estimate the likelihood of being “completely confident” in one’s ability to manage health. Key predictors included: reported use of social media to make health decisions, use of such information in clinical discussions, and the perceived difficulty distinguishing between true and false health information. The model controlled for age, sex, race/ethnicity, education, daily social media use, and self-rated health.

**Results:**

Approximately 27% of respondents reported complete health self-efficacy. Multivariable analysis showed that individuals who strongly disagreed with using social media information for health decisions were significantly more likely to report high self-efficacy (OR = 1.37, *p* = 0.041). Similarly, those who strongly disagreed that it was difficult to differentiate between true and false health information were 47% more likely to be completely confident about health management (OR = 1.47, *p* = 0.009). Using social media information in discussions with providers was not associated with health self-efficacy. Notably, women (OR = 1.57, *p* < 0.001) and daily social media users (OR = 1.38, *p* = 0.013) reported higher confidence, while Asian respondents were less likely to report being completely confident compared to their White counterparts (OR = 0.44, *p* = 0.004).

**Conclusion:**

Health self-efficacy is associated with selective non-reliance on social-media-based health information for decision making and perceived evaluative ease. These patterns are consistent with Social Cognitive Theory, which emphasizes the role of perceived competence in shaping confidence beliefs. Although causal direction cannot be established, the findings suggest that public health efforts may benefit from supporting digital health literacy and critical evaluative confidence may be relevant to strengthening confidence in health management within increasingly complex digital information environments.

## Introduction

Social media has become a common source of health information. However, less is known about how health information experiences on these platforms relate to health self-efficacy. Existing studies often measure social media use in terms of frequency or general exposure scales, which limits insight into how individuals evaluate the credibility of the health information they encounter or how they apply that information to personal and clinical decisions (1). As a result, it remains unclear whether health self-efficacy is shaped primarily by access to information, confidence in assessing its trustworthiness, or the ways in which information is ultimately used in everyday health management. The ways people encounter and engage with health information have changed fundamentally in the digital age. Rather than relying primarily on formal sources of medical information (e.g., clinicians, printed materials, institutional websites, et cetera), individuals increasingly encounter health advice, personal experiences, and medical claims through social media platforms (e.g., YouTube, WhatsApp, Facebook, et cetera), where content is created and circulated by peers, influencers, advocacy groups, and commercial and non-commercial organizations (2–4). For instance, a cross-sectional study of 374 patients found that 85% reported searching for health information on social media, with YouTube being the most frequently used platform, and most searches focused on personal or family health concerns (3).

Health content also appears frequently and often incidentally in users’ feeds, with large volumes of wellness and medical information amplified through user sharing and algorithmic recommendation systems, sometimes to the point of information overload (5–7). In a 2024 diary study of everyday social media use, 39 participants recorded 71 instances of encountering health information within 1 month, which suggested that the users were routinely exposed to health content during their everyday social media day, irrespective of any search intent (8). This suggests that social media is no longer merely a supplementary information source but a critical channel through which people are routinely exposed to and engage with health-related information.

The expansion of social-media-based health information has reshaped how health knowledge is accessed, interpreted, and shared. Digital platforms can expand access to health knowledge and support patient engagement by enabling people to seek, share, and discuss health information and experiences online (9). Prior research shows that social media can serve as a space where users encounter health promotion, community support, and peer experiences that enhance patient engagement process behaviors, particularly for groups that may face barriers in traditional information environments (10, 11). At the same time, these benefits are inseparable from the structural features of social media platforms that shape what information is visible and influential. Health information on social media is frequently produced outside traditional medical and scientific institutions, shared in short-form posts, and amplified by algorithms that prioritize engagement over accuracy (9, 12–14). For example, in an analysis of the most popular birth control-related TikTok videos, only about 10% were created by certified health professionals, with the majority produced by lay users and wellness influencers, highlighting how much health advice on social media originates outside formal expertise (15). As a result, users may encounter emotionally compelling but incomplete or unreliable information, making it difficult for users to evaluate whether information is credible or applicable to their own situation (16). In this environment, users are exposed to large volumes of health information that are both readily accessible and difficult to navigate. These challenges underscore the importance of health literacy and digital health literacy in social media environments. Health literacy supports individuals in obtaining and translating health-related knowledge in ways that are appropriate to their personal and care contexts (17). Digital health literacy adds another layer in online environments, where users must also evaluate the credibility and accuracy of information encountered through short-form posts, peer narratives, influencers, and algorithmically amplified content. Therefore, the ability to evaluate health information in digital environments is central to understanding how social-media-based health information may shape individuals’ confidence in managing their health (18).

Health self-efficacy, defined as an individual’s confidence in their ability to manage their health and carry out behaviors needed to achieve desired health outcomes (19, 20), captures how navigating complex health information environments ultimately shapes people’s sense of control over their health. This relationship can be understood through Social Cognitive Theory (SCT), which explains human behaviors as shaped by reciprocal interactions among personal factors, behavior, and the environment (21, 22). SCT suggests that besides being influenced by information environments, individuals actively interpret, evaluate, and respond to them. Health self-efficacy is therefore not simply a result of information access. It may also depend on whether individuals feel able to interpret, evaluate, trust and apply information to their own health situation. Health self-efficacy has been consistently found to be associated with medication adherence, preventive care use, chronic disease management, and health-related quality of health (23–25). In information environments characterized by high volume, variable quality, and competing claims, exposure to information alone may be insufficient to generate greater confidence and control.

Past studies examining associations between health-related social media use and health self-efficacy vary widely in the measurement of social media use. Some studies measure the frequency or intensity of engagement with health content on social media, whereas others use the composite indices to measure health-related social media use. For example, Niu et al. (26) and Mahmood et al. (27) used multi-item frequency scales to assess how often individuals read, share, or seek health information on social media and both reported positive associations between health-related social media use and self-efficacy. In Niu et al. (26), self-efficacy mediated the relationship between social media use and health behavioral intentions, with this association being stronger among individuals with more positive prior online health information experiences. In contrast, Mahmood et al. (27) identified self-efficacy as a significant predictor of preventive behavior in the context of COVID-19, without formally testing mediation. Other studies have defined social media use more broadly. For instance, Gong et al. (28) measured health-related social media use as a composite frequency-based index of multiple online behaviors and examined it as a moderating factor within a broader communication pathway, rather than as a direct predictor of self-efficacy. Similarly, Yu et al. (29) combined different types of media use and discovered that social media use was indirectly associated with health literacy through self-efficacy, with the strength of this pathway varying across populations. More recent work has moved beyond frequency-based measures to examine qualitative and experiential dimensions of social media use (30). For example, incorporated perceived information quality and emotional arousal to health content. They showed that how health information is experienced is closely related to self-efficacy. In contrast, research on problematic or excessive use have reported negative associations with self-efficacy, with measures of compulsive or addictive engagement linked to reduced confidence and poorer psychosocial outcomes (31, 32).

Despite these advances, prior studies have typically examined health information experiences either at the level of exposure or through isolated experiential constructs, often within broader mediation or moderation frameworks. Less attention has been paid to distinguishing between different stages of the health information process itself. In particular, few studies have explicitly separated how individuals evaluate health information, such as their ability to assess credibility in misinformation-prone environments, from how they act on that information, including applying it to personal health decisions or incorporating it into discussions with health care providers. As a result, it remains unclear whether health self-efficacy is shaped primarily by access to information, by confidence in evaluating that information, or by the successful application of information in personal and clinical contexts.

Building on this gap, this study focuses on how specific experiences with social-media-based health information relate to health self-efficacy. Rather than treating social media use as a single or uniform exposure, this study distinguishes between experiences related to evaluating information and experiences related to acting on information. In particular, the analysis examines whether difficulty determining the credibility of health information on social media, using social media information to guide personal health decisions, and using such information in discussions with health care providers are associated with individuals’ confidence in their ability to take good care of their health. In doing so, this study contributes to SCT-informed health communication research by clarifying how perceived capability within digital information environments may be associated with health self-efficacy. The overarching research question guiding this study is: What is the relationship between individuals’ experiences with health information on social media and their health self-efficacy?

## Methods

### Data

This study analyzed the 2022 Health Information National Trends Survey (HINTS 6), a nationally representative cross-sectional study administered by the National Cancer Institute. Since 2003, HINTS has tracked changes in the health communication and health information technology landscape and has collected nationally representative data on the U. S. public’s knowledge, attitudes, and use of cancer- and health-related information. HINTS covers a range of topics, including health information seeking, misinformation, patient-provider communication, trust in multiple information sources, health information technology, health behaviors, health care utilization, and health status. HINTS 6 surveyed how the non-institutionalized adults aged 18 years or above access and understand health information in the United States. The data was collected via mail and online between March 7, 2022, and November 8, 2022. There were 6,252 respondents, with a weighted response rate of 28.1%. Details of the survey design are available in the HINTS 6 Methodology Report (33).

### Measurements

Health self-efficacy was the dependent variable, which was measured by asking, “Overall, how confident are you about your ability to take good care of your health?” The response options were on a five-point Likert scale ranging from 1 = completely confident to 5 = not confident at all. To align with the study’s objective of identifying individuals reporting the highest level of perceived health management confidence, health self-efficacy was operationalized using a top-category approach. This study dichotomized the responses as 0 = not completely confident and 1 = completely confident. This top-category approach measures high health self-efficacy. It isolates respondents expressing maximal confidence rather than assuming equal intervals across ordinal response categories, thereby reducing nuances of some uncertainties in confidence. This approach is consistent with past studies (34, 35).

Use of health information from social media was measured using the following three statements on a four-point Likert scale ranging from 1 = Strongly agree to 4 = Strongly disagree: (1) “I use information from social media to make decisions about my health,” (2) “I use information from social media in discussions with my health care provider,” and (3) “I find it hard to tell whether health information on social media is true or false.” The third key independent variable measures perceived evaluative competence. Consistent with the operationalization of health self-efficacy as a top-category measure, responses to social-media-related items were coded to highlight individuals articulating the most definitive stance toward social-media-based health information by dichotomizing the responses as 0 = Not strongly disagree and 1 = Strongly disagree.

This study also includes control variables, such as age (1 = 18–44, 2 = 45–64, 3 = 65+), sex (0 = Male, 1 = Female), race and ethnicity (1 = White, 2 = Black, 3 = Hispanic, 4 = Asian, 5 = Other), education (0 = High school or less, 1 = Some college or more), relationship status (0 = Single, Widowed, Divorced, or Separated, 1 = Married or Cohabiting), social media use (0 = Not almost every day, 1 = Almost every day), and self-rated health (0 = Fair, or Poor, 1 = Excellent, Very good, or Good).

### Analysis

All data were analyzed using complex survey design in R version 4.5.2 to obtain population-level estimates (2). Survey weights were applied to minimize biases due to nonresponse. All variables in this study were categorical; therefore, the data was presented as frequencies and weighted percentages (%). The data was analyzed using descriptive statistics, bivariate analysis using chi-square test of independence, and the binomial logistic regression estimates of health self-efficacy. The average weighted missingness was 6.82% only. In addition, Rao-Scott adjusted chi-square tests showed no statistically significant association between missingness in health self-efficacy and the three key independent variables. Likewise, the Missingness at Random (MAR) diagnostics using logistic regression with Wald F statistics in complex survey design showed no significant association between missingness in health self-efficacy and the observed independent variables. Therefore, listwise deletion was applied, which reduced the analytical sample to 4,352. Our multivariable logistic regression model controlled for age, sex, race and ethnicity, education, relationship status, daily social media use, and self-rated health conditions. For sensitivity checks, we also ran ordinal logistic regression, and unweighted regressions.

## Results

Table 1 presents the descriptive statistics for the variables in this study. For dependent variable health self-efficacy, 1,186 respondents (26.73%) reported being completely confident about their ability to take good care of their health. As for the key independent variables, almost two-thirds of respondents (2,724; 61.24%) reported they strongly disagree that they use information from social media to make health decisions. Similarly, 2,597 respondents (59.1%) reported strong disagreement with the statement that they use information from social media in discussions with their healthcare providers. About one-sixth of respondents (710; 15.65%) reported they strongly disagree that they find it hard to tell whether health information on social media is true or false.

**Table 1:** Sample characteristics (*N* = 4,352).

Characteristic	Frequency n	Weighted %	Health self-efficacy (Rao & Scott Chi-square test, *p*-value)
Respondents reported they were completely confident about their ability to take good care of their health	1,186	26.73	
Respondents reported they strongly disagree that they use information from social media to make health decisions	2,724	61.24	<0.001
Respondents reported they strongly disagree that they use information from social media in discussions with their health care provider	2,597	59.1	0.002
Respondents reported they strongly disagree that they find it hard to tell whether health information on social media is true or false	710	15.65	0.003
Age (years)			0.221
18–44	1,524	47.42	
45–64	1,607	37.03	
64+	1,221	15.55	
Sex			<0.001
Men	1,665	48.37	
Women	2,687	51.63	
Race			0.003
White	2,482	60.54	
Black	676	11.29	
Hispanic	800	17.14	
Asian	237	6.08	
Other	157	4.95	
Education			0.097
High school or less	890	25.96	
Some college or more	3,462	74.04	
Relationship status			0.379
Single, divorced, widowed, or separated	1,972	43.47	
Married or cohabiting	2,380	56.53	
Respondents reported they use social media daily	2,872	68.76	0.027
Self-rated health is excellent, very good, or good	3,633	84.31	<0.001

As for control variables, there were 1,524 respondents (47.42%) aged 18–44; about one-third of respondents (1,607; 37.03%) aged 45–64 and just over one-fifth were 65 or above (1,221; 15.55%). In this study, female respondents (2,687; 51.63%) had slightly larger share than male counterparts (1,665; 48.37%). 2,482 respondents (60.54%) were white, 676 respondents (11.29%) were Black, 800 respondents (17.14%) were Hispanic, 237 respondents (6.08%) were Asian, and 157 respondents (4.95%) reported Other. Almost three fourth of the respondents (3,462; 74.04%) had at least some college education while 890 respondents (25.96%) had a high school education or less. Over half of the respondents (2,380; 56.53%) were married or cohabiting while 1,972 respondents (43.47%) were single, divorced, widowed, or separated. More than two third of the respondents (2,872; 68.76%) reported they use social media daily. The majority of the respondents’ (3,633; 84.31%) self-rated health were excellent, very good or good.

Table 1 also shows bivariate results from initial chi-square tests examining whether there were associations between respondents’ uses of health information in social media and high health self-efficacy. Respondents’ reports of strong disagreement for using health information from social media to make health decisions were significantly associated with high health self-efficacy (*p* < 0.001). Respondents’ reports of strong disagreement for use of health information from social media in discussions with their health care provider were associated with high health self-efficacy (*p* = 0.002). Similarly, respondents’ reports of strong disagreement for finding it hard to differentiate health information on social media being true or false were significantly associated with high health self-efficacy (*p* = 0.003).

Table 2 shows the multivariable binomial logistic regression model estimating the self-efficacy of health care. Respondents who reported they strongly disagree that they use information from social media to make health decisions were significantly more likely to be completely confident about their health care self-efficacy than those who did not strongly disagree (OR = 1.37, *p* = 0.041). Likewise, respondents who reported they strongly disagree that it is difficult for them to differentiate health related information on social media to be true or false were significantly more likely to be completely confident about their health care self-efficacy than those who did not strongly disagree (OR = 1.47, *p* = 0.009). However, respondents who reported they strongly disagree that they use health information from social media in discussions with health care providers had no association with high health self-efficacy (OR = 1.27, *p* = 0.117).

**Table 2:** Binomial logistic regression estimating the self-efficacy of health care (*N* = 4,352).

Independent variables	Odds ratio (OR)	S. E.	*p* value
(Intercept)	0.038	0.329	<0.001
Strongly disagree—use information from social media to make health decisions	1.37	0.154	0.041
Strongly disagree—use information from social media in discussions with health care provider	1.268	0.151	0.117
Strongly disagree—find it hard to tell whether health information on social media is true or false	1.474	0.149	0.009
Age (years; Ref = 18–44 years)			
45–64	0.783	0.134	0.068
64+	0.984	0.143	0.909
Women (Ref = Men)	1.566	0.128	<0.001
Race (Ref = White)			
Black	1.22	0.169	0.241
Hispanic	0.807	0.169	0.205
Asian	0.438	0.286	0.004
Other	0.804	0.292	0.456
Some college or more (Ref = High school or less)	1.087	0.15	0.577
Relationship status (Ref = single, widowed, divorced, or separated)			
Married or cohabiting	1.074	0.126	0.569
Daily social media use	1.377	0.129	0.013
Self-rated health is excellent, very good, or good	4.943	0.216	<0.001

The results showed that women were more likely to be completely confident about their health care self-efficacy than their men counterparts (OR = 1.57, *p* < 0.001). Likewise, respondents using social media daily and respondents who self-rated their health as excellent, very good, or good were more likely to report being completely confident about their health care self-efficacy (OR = 1.38, *p* = 0.013; OR = 4.94, *p* < 0.001). Although Black, Hispanic, and Other racial and ethnic groups had no significant difference in their health care self-efficacy compared to White counterparts, Asian respondents were less likely to report being completely confident about their health care self-efficacy compared to their White counterparts (OR = 0.44, *p* = 0.004). Lastly, the two control variables, education and relationship status, had no significant relationship with health care self-efficacy. The robustness check models, i.e., unweighted binomial logistic regression as well as weighted ordinal logistic regression, showed consistent results.

## Discussion

Using the data from a nationally representative 2022 HINTS 6, this study found that specific self-reported selective uses of health information from social media were significantly associated with high health self-efficacy, rather than how much they use it. Because health self-efficacy was operationalized using a top-category approach, these findings should be interpreted as association with the highest level of perceived health management confidence rather than health self-efficacy broadly. In other words, the results distinguish respondents who reported being “completely confident” in their ability to take good care of their health from other respondents, rather than estimating differences across the full range of health self-efficacy. The multivariable binomial logistic regression demonstrated that individuals who reported strong disagreement with using social media health information to make health decisions were more likely to be completely confident in their ability to manage their own health. These results may suggest that selective non-reliance on social media health information for decision making is associated with high health self-efficacy.

### Selective non-reliance on social media and high health self-efficacy

Consistent with the foundational tenets of self-efficacy theory, which posits that efficacy beliefs operate within specific domains, rather than as a global personality trait, these behaviors represent a targeted application of confidence to the health domain (30, 36, 37). Although broader literature suggests that general self-efficacy is a primary predictor of information-seeking intentions (26, 38), our results suggest a possible interpretation that individuals with high level of perceived health management confidence may take a more cautious approach to evidence. Individuals who report not relying on social media when making health decisions may be describing a more selective and cautious engagement with social-media-based health information.

As established in Deng and Liu (38)‘s model, self-efficacy may shape how individuals respond to perceived risk on intention, leading individuals to take a more scrutinizing approach to safeguard their outcomes. This motivation to scrutinize is further explained by meta-analytical findings showing that when outcomes feel personally important or “threatening,” individuals are more motivated to move beyond anecdotal social media content and seek higher informational standards. By self-reporting non-use of social media for health decisions, these individuals describe an informational environment they perceive as manageable, which is strongly associated with the highest level of health self-efficacy in this study.

However, this interpretation should be viewed as one possible explanation rather than a definitive conclusion. Selective non-reliance should not be viewed as inherently superior behavior or a direct driver of high health confidence. It is best understood as a self-reported, context-dependent pattern of information use that may reflect a preference for trusted alternatives, such as health care providers, institutional websites, or family members. Future studies should examine how individuals combine, substitute different health information sources to make health decisions and how they are associated with health self-efficacy.

### Perceived evaluative competence and high health self-efficacy

This study showed a significant association between the perceived ease of differentiating between true and false health information on social media and the highest level of health self-efficacy. Respondents who strongly disagreed that it was difficult for them to evaluate the veracity of social media content were significantly more likely to report being completely confident in their health management. This suggests that perceived evaluative competence that is, that is, respondents’ self-perceived ability to recognize the limitations of social media content—may be consistent with a perceived sense of mastery among individuals reporting the highest level of perceived health self-efficacy. It is critical to note, however, that this study measures self-perception; respondents may overestimate or underestimate their ability to discern misinformation, and complete confidence does not necessarily equate to factual accuracy. Accordingly, this measure is interpreted as perceived evaluative capacity rather than objective digital literacy.

According to SCT, mastery experience (successful performance on a task) is an influential source of self-efficacy because it provides authentic evidence of capability (21, 39, 40). In the modern digital landscape, the perceived ability to reject unreliable or anecdotal information constitutes a form of perceived mastery. When individuals believe they are successfully navigating a complex informational environment, this belief, even if it does not necessarily reflect actual evaluative competence, may reinforce their belief in their ability to take good care of their health. Conversely, for those who find evaluation difficult, the cognitive burden and potential exposure to misinformation may undermine their confidence in taking care of their health (41, 42). For these less-confident groups, practitioners may help individuals achieve small, successful milestones in health management and information evaluation. Improving perceived evaluative competence may support a foundational sense of control and confidence in health management.

It is worth noting that the highest-level health self-efficacy does not appear to be driven by avoidance of social media, as daily social media users were more likely to report high confidence. Frequent users may perceive the platform as a domain to continuously exercise and validate their evaluative skills.

### Socio-demographic disparities in high health self-efficacy

The study also highlights significant demographic variations in the association between social media experiences and reporting of extreme/high health self-efficacy. Women were significantly more likely to be completely confident in their health care self-efficacy than their male counterparts, which aligns with research where women adopt proactive, caregiving-like roles in self-management and men may report lower self-care confidence (43). Furthermore, self-rated health was associated with those in excellent, very good, or good health reporting vastly higher confidence, consistent with previous work finding that better perceived health status closely tracks higher self-efficacy (44, 45). These suggest that health management confidence is tied to both current health status and gendered approaches to caretaking. Notably, Asian respondents were significantly less likely to report being completely confident in their health management compared to White counterparts. This may reflect cultural factors, language, immigration history, health literacy, health care access, or institutional barriers may affect the development of health management confidence. For these communities, as well as for men and those in fair or poor health, interventions may consider focusing on building institutional trust and providing authoritative, vetted digital resources that are culturally and linguistically tailored to build genuine navigation mastery. By supporting successful navigation of the digital health information environment, public health efforts may be relevant to helping strengthen health management confidence in these groups. Since our models do not explicitly measure the mechanisms behind this racial difference in health care self-efficacy, future studies can examine and identify those mechanisms.

Our study has several limitations, so it is important to interpret the results of this study within the context of these limitations. The cross-sectional nature of the HINTS 6 data precludes definitive conclusions regarding causality. Although we examined social media experiences as the key independent variables, the relationship between extreme/high health self-efficacy and behavior on social media may be bidirectional. On one hand, perceived mastery in evaluating health information in social media may influence complete confidence in health management. On the other hand, individuals with high health self-efficacy may be more selective in how they use social media health information or may perceive themselves as more capable of evaluating its credibility. Because the data are cross-sectional, this study cannot determine the temporal ordering of these pathways. Furthermore, all measures were based on self-reported data, which may be subject to social desirability bias or an overestimation of one’s perceived evaluative skills. The item on distinguishing true from false health information on social media captures perceived evaluative ease rather than validated misinformation detection ability or actual digital health literacy. Additionally, the dichotomization strategy in survey responses may obscure gradations in confidence and social media engagement; therefore, findings reflect differences between the highest-confidence subgroup and all other respondents rather than linear or ordinal associations across the full health self-efficacy scale. Lastly, listwise deletion for logistic regression generally gives valid inferences under broader conditions, but since it did not use all of the available information, there was still some risk of bias. Therefore, future studies should consider handling missing data for categorical variables using a more robust approach such as Full Information Maximum Likelihood (FIML). Future studies should employ longitudinal designs to establish the temporal ordering between social media engagement and different levels of health self-efficacy, clarifying whether selective non-reliance on social media for health decisions predicts health management confidence. Furthermore, studies should incorporate objective, performance-based assessments of digital literacy to validate self-reported evaluative skills and ensure that high health self-efficacy is grounded in actual competence rather than perceived mastery.

## Conclusion

This study contributes by distinguishing between reported reliance on social-media-based health information and perceived evaluative difficulty within a nationally representative sample from HINTS 6. Our findings suggest that selective non-reliance on social-media-based health information for decision making and perceived evaluative ease are associated with high health self-efficacy. The results demonstrate that individuals who report avoiding reliance on social media content specifically those who do not rely on it for health decisions and those who feel confident in their ability to discern truth from false content report significantly higher health self-efficacy. In the context of Social Cognitive Theory, the ability to navigate and avoid unreliable digital content may reflect a mastery experience. Such perceived evaluative ability is consistent with greater confidence in managing one’s health, although temporal direction cannot be established. Conversely, we found no significant relationship between using social media information in discussions with healthcare providers and health self-efficacy. This suggests that the “action” of bringing digital information into the clinical setting may not be independently associated with a patient’s sense of self health-management as much as the internal “evaluation” of that information does.

As digital platforms increasingly rely on algorithms that prioritize engagement over factual accuracy, the ability to distinguish credible from misleading health information becomes a prerequisite for effective health management. Although social media offers opportunities for community support, perceived difficulty in navigating health information on these platforms is associated with lower confidence in health self-management. Therefore, public health interventions need to move beyond passive fact-checking and instead provide proactive digital navigation toolkits that help patients identify the structural markers of credible health content on social media in an increasingly complex digital environment.

## Data Availability

Publicly available datasets were analyzed in this study. This data can be found at: https://hints.cancer.gov/data/Default.aspx.
